# A device for assessing microbial activity under ambient hydrostatic pressure: The in situ microbial incubator (ISMI)

**DOI:** 10.1002/lom3.10528

**Published:** 2022-12-14

**Authors:** Chie Amano, Thomas Reinthaler, Eva Sintes, Marta M. Varela, Julia Stefanschitz, Sho Kaneko, Yoshiyuki Nakano, Wolfgang Borchert, Gerhard J. Herndl, Motoo Utsumi

**Affiliations:** ^1^ Department of Functional and Evolutionary Ecology, Bio‐Oceanography Unit University of Vienna Vienna Austria; ^2^ Instituto Español de Oceanografía‐CSIC, Centro Oceanográfico de Baleares Palma de Mallorca Spain; ^3^ Instituto Español de Oceanografia‐CSIC, Centro Oceanografico de A Coruña A Coruña Spain; ^4^ NiGK Corporation Saitama Japan; ^5^ Japan Agency for Marine‐Earth Science and Technology (JAMSTEC) Yokosuka Japan; ^6^ Briese Schiffahrts GmbH & Co. KG Leer Germany; ^7^ NIOZ, Department of Marine Microbiology and Biogeochemistry Royal Netherlands Institute for Sea Research, Utrecht University Texel The Netherlands; ^8^ Faculty of Life and Environmental Sciences University of Tsukuba Ibaraki Japan; ^9^ Microbiology Research Center for Sustainability University of Tsukuba Ibaraki Japan; ^10^ Present address: Marine Evolutionary Ecology, Deep‐Sea Biology Group, GEOMAR Helmholtz Centre for Ocean Research Kiel Kiel Germany

## Abstract

Microbes in the dark ocean are exposed to hydrostatic pressure increasing with depth. Activity rate measurements and biomass production of dark ocean microbes are, however, almost exclusively performed under atmospheric pressure conditions due to technical constraints of sampling equipment maintaining in situ pressure conditions. To evaluate the microbial activity under in situ hydrostatic pressure, we designed and thoroughly tested an in situ microbial incubator (ISMI). The ISMI allows autonomously collecting and incubating seawater at depth, injection of substrate and fixation of the samples after a preprogramed incubation time. The performance of the ISMI was tested in a high‐pressure tank and in several field campaigns under ambient hydrostatic pressure by measuring prokaryotic bulk ^3^H‐leucine incorporation rates. Overall, prokaryotic leucine incorporation rates were lower at in situ pressure conditions than under to depressurized conditions reaching only about 50% of the heterotrophic microbial activity measured under depressurized conditions in bathypelagic waters in the North Atlantic Ocean off the northwestern Iberian Peninsula. Our results show that the ISMI is a valuable tool to reliably determine the metabolic activity of deep‐sea microbes at in situ hydrostatic pressure conditions. Hence, we advocate that deep‐sea biogeochemical and microbial rate measurements should be performed under in situ pressure conditions to obtain a more realistic view on deep‐sea biotic processes.

Microbes in the dark ocean play a key role in the ocean's biogeochemical cycling and are responsible for approximately half of the microbial heterotrophic carbon production in the global ocean (Aristegui et al. 2009). It is well established now that prokaryotic activity and prokaryotic community composition are depth‐stratified in the oceanic water column, mainly in response to temperature (Lonborg et al. 2016; Moran et al. 2017) and substrate availability (Guerrero‐Feijoo et al. 2017; Varela et al. 2020; Rodriguez‐Ramos et al. 2021) with carbon and nitrogen driving changes in microbial community composition (Mende et al. 2017). The few studies measuring deep‐sea prokaryotic activity under in situ pressure conditions suggest that hydrostatic pressure strongly influences prokaryotic activity by either decreasing or increasing the rates measured under pressurized conditions (Jannasch et al. 1976; Jannasch and Wirsen 1982; Deming and Colwell 1985; Bianchi and Garcin 1994; Tholosan et al. 1999; Tamburini et al. 2002, 2003, 2009; Garel et al. 2019). The vast majority of studies measuring deep‐sea microbial activity, however, have been performed under atmospheric pressure conditions (Aristegui et al. 2009; Herndl et al. 2022).

The discovery of pressure‐adapted piezophilic isolates from deep‐sea sediments of the Philippine and Kermadec‐Tonga Trench spurred the debate on the effects of pressure on deep‐sea microbial activity (Zobell and Morita 1957). In the early days of deep‐sea microbiology, samples were taken with equipment not able to maintain the hydrostatic pressure during recovery and the samples were simply repressurized on board the vessel. In later attempts to measure in situ activity, the submersible Alvin was used to manipulate samples on the seafloor (Jannasch and Wirsen 1973). Simultaneously, various types of pressure retaining bottles were developed (Gundersen and Mountain 1972; Jannasch et al. 1973; Tabor et al. 1981), allowing to sample seawater at any depth and keeping the sample pressurized upon retrieval. These types of samplers are usually composed of a metal cylinder and high‐pressure valves that are opened and closed at depth with a variety of mechanisms to collect seawater without the help of external pumps and complex electronics. While controlling the hydrostatic pressure and preventing potential pressure loss during the recovery can be technically challenging, however, more advanced pressure retaining instruments are in use today (Kato et al. 2008; Zhang et al. 2015; Garel et al. 2019; Peoples et al. 2019). In addition, recently developed laboratory pressure chambers provide insights into prokaryotic activity under deep‐sea hydrostatic pressure conditions (Wannicke et al. 2015; Stief et al. 2021).

An alternative approach for measuring prokaryotic activity under hydrostatic pressure conditions is incubating the samples in situ (Seki and Robinson 1969). Compared to pressure retaining devices which are hoisted on deck of the ship for further analyses, sampling containers of in situ incubators can be made of material other than metal (e.g., polycarbonate, polyethylene, polypropylene, polytetrafluoroethylene [PTFE], acrylyl). Most of the commercially available plastic material tested and used for incubations in the lab are potentially suitable for application in the dark ocean and thus, technical difficulties concerning design and machining of metals are reduced and production costs are significantly cut. More importantly, coated stainless steel or titanium (generally used for pressure retaining samplers) are more difficult to clean than, for example, polycarbonate vessels.

An in situ incubator with a long development history is the submersible incubation device (SID), initially developed to measure euphotic zone primary production based on ^14^C‐bicarbonate incorporation (Taylor et al. 1983; Taylor and Doherty 1990; Taylor et al. 1993). Recently, modified versions of the SID have been used for estimating nitrogen fixation in the upper 100 m of the North Pacific (Bombar et al. 2015), for experiments with protists grazing on prokaryotes at depth down to 3500 m in the Mediterranean (Pachiadaki et al. 2016) and transcriptome analysis of bathypelagic samples compared to Niskin bottle samples (Edgcomb et al. 2016). There are several other large instruments available for sampling, incubation, filtration, preservation and analysis of microbes at depth (reviewed in McQuillan and Robidart 2017). However, a small and flexible incubator that allows autonomous sampling, incubation and fixing of replicate seawater samples down to the base of the bathypelagic waters has not been described yet.

Here we describe an in situ microbial incubator (ISMI) that allows to run incubations from sampling to fixation down to a depth of 4000 m. Depending on the configuration, the instrument incubates volumes from 50 mL to 10 L with up to six replicates. Particularly small volumes (10–100 mL) are used in incubations with radiolabeled or other model substrates for prokaryotic activity measurements, such as ^14^C‐glucose, ^3^H‐thymidine, ^14^C‐bicarbonate, and ^14^C‐ or ^3^H‐leucine (Parsons et al. 1984; Chin‐Leo and Kirchman 1988; Kirchman 2001), 5‐bromo‐2′‐deoxyuridine (BrdU) and 5‐ethynyl‐2′‐deoxyuridine (EdU) (Steward and Azam 1999; Smriga et al. 2014) and l‐azidohomoalanine (AHA) and l‐homopropargylglycine (HPG) (Samo et al. 2014; Hatzenpichler and Orphan 2015). The small size and weight of the instrument allows different ways of deployment, for example, attached to a wire, a CTD rosette frame or a freely floating buoy. All the materials in contact with the sample are made of biologically inert plastic that is easy to clean and decontaminate. The design and electronics are intentionally kept simple allowing scientists to run and maintain the system without an additional technician on board of research vessels. Any radiolabeled or model substrate applied to estimate prokaryotic activity in seawater can be used.

In this study, the performance of the ISMI has been evaluated by measuring ^3^H‐leucine incorporation rates of prokaryotes (Kirchman et al. 1985), a method that is both, very sensitive to all sorts of contamination and easy to compare to protocols commonly applied in many labs. We describe the different tests to verify the proper functioning of the ISMI. In addition, we present prokaryotic heterotrophic activity measurements performed in the deep sea under in situ hydrostatic pressure conditions and compare them to those performed following decompression and incubation at atmospheric pressure conditions.

## Materials and procedures

### Function of the ISMI

**Fig. 1:**
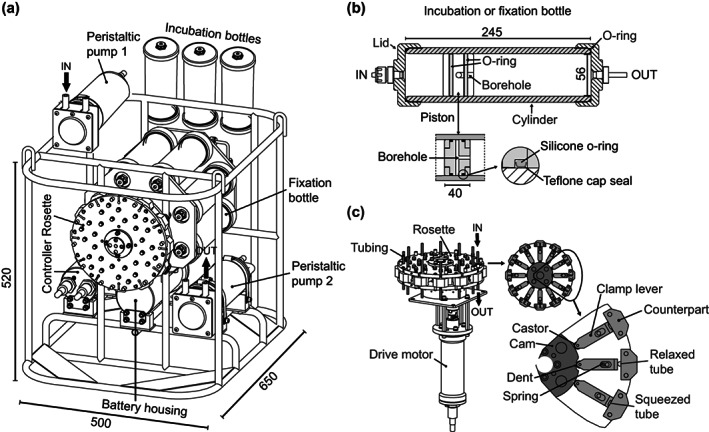
(**a**) Overview of the components of ISMI. Silicone tubing connects the individual components. (**b**) Assembled sampling bottle. Sectional view of an ISMI sampling bottle. A hole in the piston allows collecting the water in‐between two O‐rings under compressed condition. The silicone O‐rings are cap‐sealed with teflon for smooth piston movement. (**c**) Rosette tubing clamp unit driven by a motor. Enlarged image of the rosette tubing clamp shows how to open and close the line with the clamps.

**Fig. 2:**
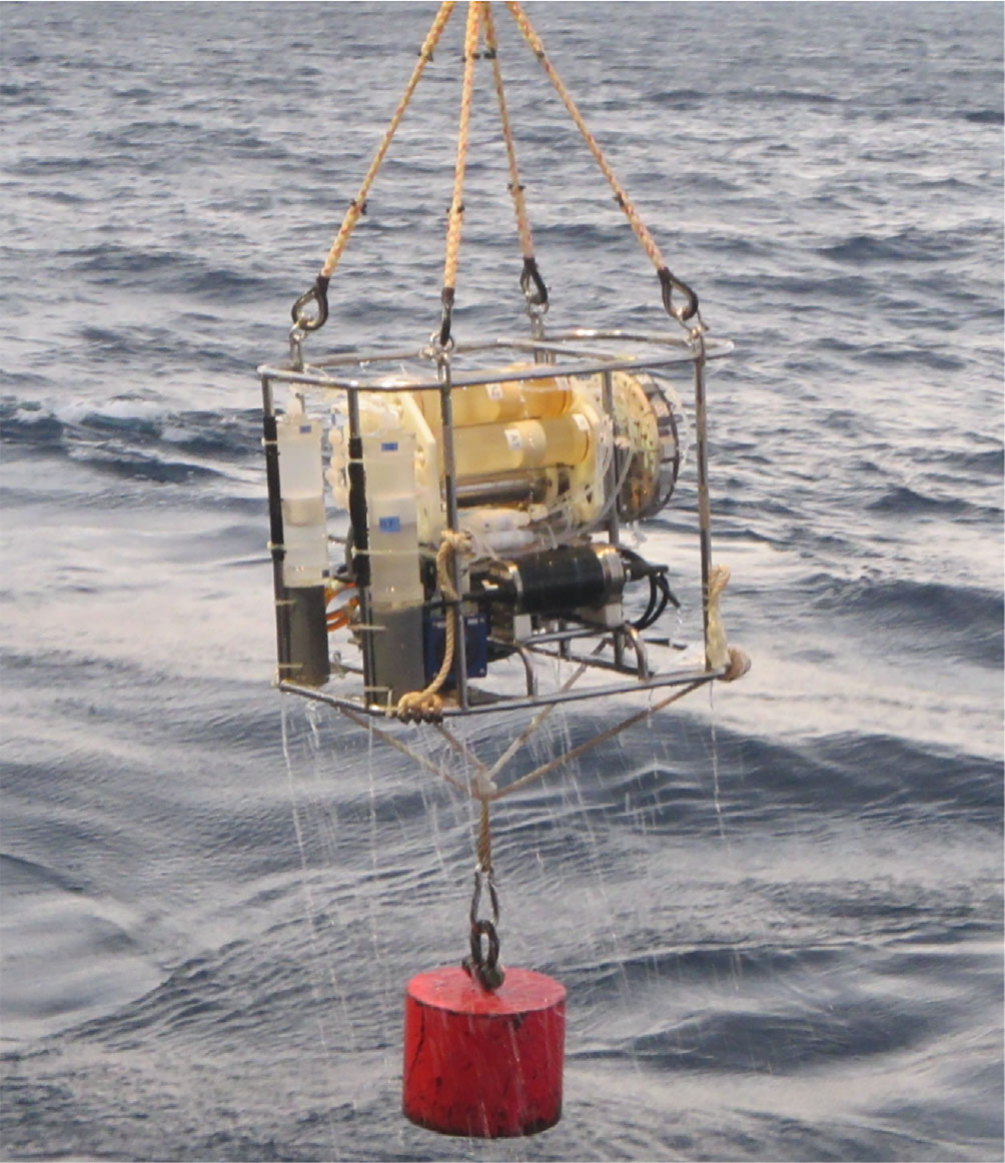
Deployment of ISMI with a winch cable installed at the vessel.

The ISMI was built by Nichiyu Giken Kogyo (NiGK) Corporation, Japan, by modifying a rotary clean seawater sampler (ROCS) which has been used for collecting samples from hydrothermal vents (Biddle et al. 2014). The ISMI consists of two peristaltic pumps, up to nine bottles for incubations and storing fixed samples, a tubing clamp rosette including a driver motor, an electronics controller and a battery housing attached to a stainless steel frame (Fig. 1a). The empty system weighs 44 kg and all parts are rated to a pressure corresponding to 4000 m depth. The ISMI can be attached to any winch‐driven cable or mounted on a CTD‐frame for deployment (Figs. 2, S1a,b).

The primary peristaltic pump transfers surrounding seawater through check valves into three sampling bottles serving as incubation chambers of live samples (Figs. 1b, S2). A time‐controlled rosette with tubing clamps distributes samples coming from the incubation chambers over up to six additional bottles used for subsampling and fixation of live samples. The tubing clamp setup is preferred over metal valves to avoid contamination of samples. A series of clamp levers sits on a clockwise rotating disc and sequentially squeezes and releases the connection tubing, thereby stopping or allowing the flow of seawater between bottles (Figs. 1c, S3). A second peristaltic pump draws the seawater from the incubation chambers to the fixation bottles. Both pumps in the ISMI deliver between 110 and 170 mL seawater min^−1^ (depending on the battery level). The minimum sampling volume with the peristaltic pump is ~ 3 mL. Accuracy of the sampling volumes was checked by comparing the programed volume to the collected volume of samples and was generally within < 5% of the programed volume.

**Fig. 3:**
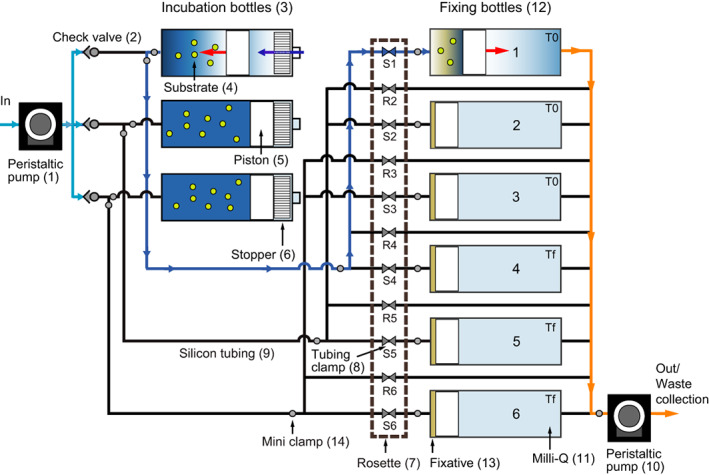
Schematic overview of flow line of ISMI. Peristaltic‐pump (1) collects ambient seawater to the incubation bottles (3) through check valves (2) (light blue lines) and mixed with substrate added prior to the deployment (4). Piston (5) can move in both directions. It stops moving when it reaches the stopper (6). Right after filling the seawater in the incubation bottles, the sample in the first incubation bottle is transferred to the first fixation bottle (12) and mixed with the fixative (13) (dark blue line). Another peristaltic pump (10) pushes the milli‐Q water (11) to the outside (orange line). The fixation bottles from No. 1 to 6 collect samples in this order regulated by the tubing clamp (8) mounted on a rosette (7). Hence, with the triplicate sampling bottles, No. 1, 2, and 3 are T0s, and No. 4, 5, and 6 are Tfs. Rinsing steps with sample seawater are performed prior to collecting the sample into the fixation bottles. Those parts are connected by silicone tubing (9). Mini clamps (14) are used to close the line while preparing ISMI and after ISMI has arrived on deck.

All vessels for incubation and fixation are made of polycarbonate and can hold a maximum volume of 500 mL. Both ends of the vessels can be closed with fitting screw caps (Fig. 1b). A movable piston made from inert PTFE (Fig. 1b) inside the bottles is used to dampen the filling speed and to push seawater out of the bottle. The collected volume in the incubation bottles can be adjusted with rolled‐up plastic sheets of 0.5‐mm thickness. In addition, there is a second set of polycarbonate bottles with a maximum volume of 250 mL. The incubation and fixation bottles are connected by silicone tubing with an inner diameter of 3 mm and an outer diameter of 6 mm. To avoid sudden pressure excursions and squeezing of air‐filled spaces inside the tubing or the bottles, the back side of the piston is filled with Milli‐Q water while the connection tubing is filled with 0.2‐μm filtered seawater (Fig. 3). Since the incubation bottles do not need a specialized holder and tubing connections are flexible, it is possible to vary the number of bottles and types of incubation containers. For large incubation volumes beyond 500 mL, the incubation bottles can be replaced by 10 L plastic folding bags (low density polyethylene, Union Container, AS ONE, Japan; Fig. S1c). By changing some tubing connections, up to three subsamples per bag can be collected over time from duplicate incubation bags. A 12 L titanium tank (NiGK; Fig. S1d,e) is optional for high temperature conditions. In general, the moveable parts in direct contact with water samples do not require grease and, including the tubing and all other connectors, they can be thoroughly cleaned with 0.5 N HCl and autoclaved.

The pumps and the driver motor of the tubing clamp rosette are powered by a 24 V rechargeable lithium‐ion pack (≤ 100 Wh, IKS Japan) or 16 commercially available D‐cell alkaline batteries. The minimum voltage needed to run the system is 12 V. Consumption over 8 h of incubation with a total of 5 L of pumped water at 2–4°C is usually < 1 V. The maximum duration of a deployment is around 1 month.

A sampling schedule with starting time for the pumping events and rotation of the tubing clamp rosette is preset with a custom‐made software (noncommand line interface software named as N‐Com communicator, ROCS‐Com). The programming scheme is simply following the software's instruction by answering questions and entering the data in the pop‐up window with a prepared setting sheet (Fig. S4a). The preprogramed time to collect water and fix the samples is transmitted to the controller on the ISMI with a RS‐232 cable connection (Fig. S4b). The controller unit has an additional button battery to supply energy to an internal clock and to generate a log file including information on date, daytime, tubing clamp rosette position and sample volume collected at each position. Time synchronization of the internal clock with the time given by the PC is always required prior to the deployment.

### Operating scheme of ISMI

Prior to the deployment, radiolabeled substrate and the fixative reagent are added to the inlet of the incubation and fixation bottles, respectively (Fig. 3). Once the ISMI is at the target depth, the first peristaltic pump pushes surrounding water through the PTFE one‐way check valves into the incubation bottles where the samples mix with the substrate. After the bottles are filled, a programable volume of each of the three incubation bottles is drawn through successively opening ports of the tubing clamp rosette into the fixation bottles with a second peristaltic pump. The check valves installed after the first pump prevent the outflow of sample through the primary inlet tube. In the fixation bottles, the samples are mixed with fixative and serve as killed controls for the live incubations. The remaining live samples are incubated for a certain period of time and subsequently fixed as described above. Prior to any transfer of samples to the fixation bottles, the connection tubing is automatically rinsed with the sample to prevent cross‐contamination between samples.

### Measurement of heterotrophic prokaryotic activity

Heterotrophic prokaryotic activity was determined via ^3^H‐leucine incorporation following the method described in Reinthaler et al. (2010) with some modifications. Prior to the deployment of ISMI, seawater from the intended depth of sampling was collected with Niskin bottles and filtered through 0.2‐μm polycarbonate filters (Nuclepore, Whatman) to set up the ISMI before deploying (see below).

Fresh Milli‐Q water was filled into the back‐end of the incubation and fixation bottles using a squeezing bottle. Filtered 37% formaldehyde was injected into the fixation bottles with a 10 mL syringe. The volume was adjusted to reach a final concentration of 2% formaldehyde. Density adjusted ^3^H‐leucine (PerkinElmer) with a specific activity of about 120 Ci mmol^−1^ was injected into the silicone tubing and connected to the inlet of the incubation bottles. The injection volume was adjusted to reach a final concentration of 5 or 10 nmol L^−1^ leucine, depending on the expected activity of the sample. All silicone tubings were filled with filtered seawater (according to the procedure described above) from the intended deployment depth of the ISMI before connecting them to the bottles and pumps as well as the ports of the tubing clamp rosette. Mini‐tubing clamps (Bel‐Art Scienceware) were used to temporarily close the silicone tubing.

The sampling and incubation schedule was programed including the time of collecting seawater at the depth of sampling, the time for fixing the samples at the beginning (T0) and the end (Tf) of the defined incubation time and the volume of seawater used for rinsing the tubing in‐between subsampling. The duration of incubation was chosen depending on the depth and varied between 3 and 15 h. For meso‐ and bathypelagic waters, we sampled 500 mL into each incubation bottle and 100–200 mL subsamples each were used for the control fixed with formaldehyde right after the sample was mixed with the tracer. The live samples were fixed at the end of the incubation period. A volume of 50 mL was reserved for rinsing the tubing (three times the tubing volume). A high‐accuracy pressure transducer was attached to the ISMI to log the depth during the incubation. To ensure a stable position of the ISMI in the water column, a weight (~ 50 kg) was attached at the end of the cable (Fig. 2).

After recovery of the ISMI, the mini‐tubing clamps were closed to prevent leakage of collected water during detaching the bottles (Fig. 3). All samples fixed at depth were transferred to 50 mL conical tubes (Greiner Bio‐One) using 50 mL syringes and stored in the lab at 4°C until further processing. The collected sample volume of each fixation bottle was measured, since the collection volume of the sample is influenced by the accuracy of the peristaltic pump (< 5%). To compare prokaryotic leucine incorporation rates between in situ and atmospheric pressure, seawater was collected with Niskin bottles mounted on a CTD rosette at the same depth as the ISMI was deployed. Three live samples and two formaldehyde‐killed controls were transferred to spare bottles of the ISMI and incubated on board at in situ temperature in the dark. The concentration of the added leucine and fixative as well as incubation time were the same in both, on board and in situ.

All samples collected were filtered onto 0.2‐μm polycarbonate filters (25‐mm filter diameter, GTTP, Millipore; 0.45‐μm support filters, HWAP, Millipore) and washed twice with 5% ice‐cold trichloroacetic acid for 5 min. Subsequently, the filters were placed in scintillation vials. Eight milliliters of scintillation cocktail (Filter Count; PerkinElmer) was added to the vials and after ~ 16 h the filters were analyzed in a liquid scintillation counter (Tri‐Carb; Packard/PerkinElmer).

Cleaning the parts of the ISMI in contact with seawater samples is critical for the quality of the measurements. All the bottles, tubes and connectors were stored in ~ 0.5 N HCl overnight and shortly before mounting them on the ISMI, they were rinsed with copious amounts of Milli‐Q water and subsequently, with 0.2‐μm filtered seawater. Material in contact with formaldehyde was washed separated from all other parts to minimize the potential of contamination with the fixative.

## Assessment

### Comparison between ISMI incubation bottles and commercially available containers

To compare the performance of the custom‐made sampling bottles of the ISMI to other material more frequently used for incubations, experiments were conducted with surface samples from the northern Adriatic Sea (on three occasions) and a bathypelagic sample from the Atlantic Ocean. Surface seawater was collected from a running seawater tank at the Ruđer Bošković Institute for Marine Research (Croatia) on two consecutive days in October 2014 and bathypelagic water was sampled with Niskin bottles at ~ 3000 m at Sta. A3 during the M139 cruise in July 2017 (Table S1). We used the ISMI polycarbonate bottles including the piston made of PTFE, commercially available 50 mL polypropylene centrifuge tubes (Cat.Nr. 227261; Greiner Bio‐One), polycarbonate media bottles with 125 mL volume (Cat.Nr. 2015‐0125; Nalgene), 50 mL bags (CX5‐14 film, inner side: low density polyethylene, sterile, Labtainer BPC Bag, Thermo Fisher Scientific) and 60 mL glass vials. In all containers, 50 mL of ^3^H‐leucine‐spiked seawater samples were incubated. The incubations and further treatment of samples were according to the procedure described above.

Overall, the measured leucine incorporation rates amounted to 200–500 and ~ 0.1 pmol leu L^−1^ h^−1^ in the surface and deep‐water samples, respectively (Table 1). There was no significant difference between different incubation materials in the deep samples (Kruskal–Wallis rank sum test, *n* = 8, *p* = 0.127). In the surface water samples, sampling dates affected the leucine incorporation rates more than the materials used as incubation vessels (two‐way ANOVA Type II, *n* = 23, *p* = 1.9 × 10^−15^ for the sampling date, *p =* 0.001 for the material). ISMI bottles resulted in similar leucine incorporation rates as the polycarbonate media bottles (Tukey–Kramer test, *p* = 0.61), whereas higher rates were obtained in the polypropylene centrifuge tubes than in the polycarbonate and the ISMI bottles (Tukey–Kramer test; *p* = 0.012 and *p* = 0.001, respectively). Commercially available bags were tested only once, yet, there was no significant difference among the bag and the other materials (Kruskal–Wallis rank sum test, *n* = 9, *p* = 0.096). Thus, our results indicate that the sampling bottles used for the ISMI yield results not significantly different from commercially available incubation containers.

**Table 1:** Bulk leucine incorporation rates (pmol leu L^−1^ h^−1^) obtained in the different incubation containers. Samples were collected in the coastal northern Adriatic Sea (surface) and in the Atlantic Ocean during the M139 cruise (deep). Number of samples is indicated in brackets. Experiments with surface water were conducted on three occasions. The deep sample was kept at 4°C for 5 d prior to the incubation. ND, not determined; temp inc., incubation temperature; duration, duration of incubation.

Sample	Sampling date	Depth (m)	Temp. in situ (°C)	Temp inc. (°C)	Duration (h)	ISMI bottle	Centrifuge tube	Media bottle	Bag	Glass vial
Surface	24 Oct 2014	20	19.5	19.5	2.2	251 ± 18 (2)	272 ± 5 (4)	239 ± 40 (2)	ND	ND
	24 Oct 2014		19.7	19.7	2.4	341 ± 13 (2)	373 ± 8 (4)	357 ± 13 (2)		
	25 Oct 2014		19.5	19.3	2.3	470 ± 9 (2)	532 ± 16 (3)	493 ± 28 (2)	491 ± 21 (2)	
Deep	22 Jul 2017	3000	2.7	2	12–13	0.12 ± 0.01 (4)	0.12 ± 0.01 (2)	ND	ND	0.11 ± 0.00 (2)

### Distribution of the substrate in ISMI bottles

The distribution and mixing of the substrate with the sample water in the bottles was tested with a dye‐colored solution and artificial seawater (ASW, salinity 35, density: 1.03 g cm^−3^ at 20°C). The dye‐solution was prepared with a near identical density to the ^3^H‐leucine working solution used in this study. Food‐dye with ~ 5% food coloring (Städter, Germany) was dissolved in ASW and 2% ethanol (to include the original solvent of the radiolabeled leucine stock solution). NaCl was used to adjust the density of the dye‐solution between 1.00 and 1.04 g cm^−3^ at 20°C.

At the start of the experiment, the dye‐solution was injected into a silicone tubing connected to the inlet of an incubation bottle. The peristaltic pump, set at 50 mL min^−1^ which is the typical flow rate for filling the three incubation bottles in situ, pumped the ASW into the bottle. The homogenous distribution of the dye was visually inspected. A difference in density larger than ~ 0.01 g cm^−3^ caused the dye‐solution to float on top of the ASW or sink (Fig. S5). A homogenous distribution of the substrate was obtained with a dye‐solution density of 0.002–0.003 g cm^−3^ lower than the ASW (Fig. S5).

Following these tests in the laboratory, an experiment was conducted with bathypelagic seawater collected during the RADCAN201808 cruise in the North Atlantic (Table S1; Sta. G4). Sample seawater was kept at 4°C for 6 d and subsequently at 17°C overnight prior to the experiment. Five ISMI incubation bottles were placed in a temperature‐controlled room at 17°C. ^3^H‐leucine was adjusted to a density of 0.0025 g cm^−3^ lower than that of the sampled seawater with NaCl (previously combusted in a muffle oven at 450°C for 2 h) and injected into a tube connected to the inlet of a bottle (final conc. 5 nmol L^−1^). All bottles were filled with 500 mL of seawater using a peristaltic pump (filling speed 50 mL min^−1^) which was then mixed with the leucine solution from the inlet by pumping without any additional disturbance. One bottle was thoroughly rotated to ensure complete mixing. From two bottles, 250 mL were immediately removed to check for premature loss of leucine due to subsampling. The removed seawater was collected in 50 mL centrifuge tubes (Greiner Bio‐One) to measure the actual radiolabeled leucine concentration calculated from a 10 μL subsample from the tubes. In the incubation bottles, no additional turbulence was applied. After a 12‐h incubation period, the seawater was gently collected into the centrifuge tubes and fixed with filtered formaldehyde. The first and last fractions as well as a few intermediate fractions were used to determine the leucine concentrations in the water and leucine incorporation rates. A sample fixed at the beginning of the incubations served as formaldehyde‐fixed control for all samples. In the mixed control, two fractions (inlet and outlet side of the bottle) were collected to measure leucine concentrations and incorporation rates.

**Fig. 4:**
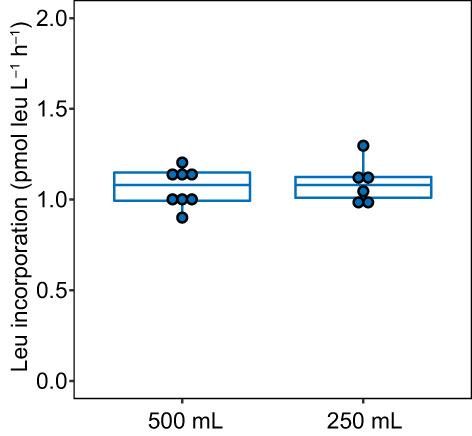
Leucine incorporation rate in detached ISMI bottles determined with fractions from full 500 mL (*n* = 8) and subsampled 250 mL bottles (*n* = 6).

Leucine incorporation rate in the mixed bottle was 1.15–1.17 pmol L^−1^ h^−1^ with < 1% difference in the leucine concentration. Over all the fractions collected from the bottles without additional mixing, concentrations of leucine were similar except a fraction from the side closest to the inlet of the bottle (0.1–0.9 nmol L^−1^ lower than the other fractions). Practically, the first ~ 50 mL seawater samples are used for rinsing the incubation system (Fig. 3). Thus, the leucine concentrations in the incubation system averaged 4.8 ± 0.1 nmol L^−1^ (mean ± SD, *n* = 11). In addition, there was no significant difference between the leucine incorporation rates collected from the 500 mL, the subsampled 250 mL and the thoroughly mixed bottle (Fig. 4; Kruskal–Wallis rank sum test, *n* = 16, *p* = 0.446). Taken together, these experiments indicate that a density of the substrate slightly lower than the ambient seawater ensures complete mixing and a homogenous distribution of the radiolabeled substrate in the collected seawater.

### Testing for potential biases due to the ISMI setup

**Fig. 5:**
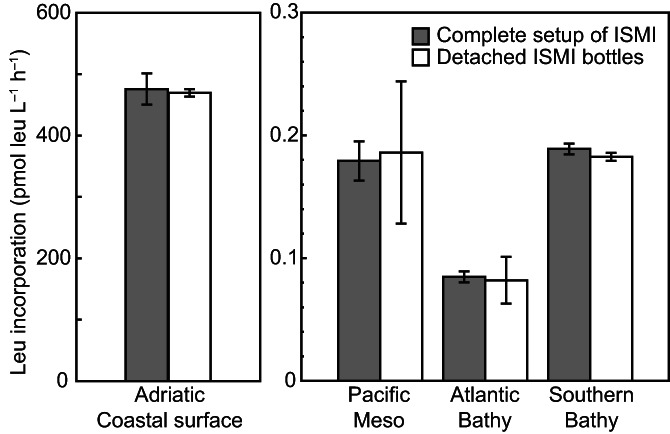
Bulk leucine incorporation rates in surface and deep seawater incubated under atmospheric condition either with the complete setup of ISMI or detached ISMI sampling bottles. Incubations were conducted in duplicate for the surface sample and in triplicate for the deep sample. Error bars indicate |mean − replicate| (*n* = 2) for the Adriatic and SD (*n* = 3) for the other samples. Meso, mesopelagic; bathy, bathypelagic water.

We checked for potential biases in rate measurements caused by the pump, tubing material and initial dilution of sample with filtered seawater at atmospheric pressured conditions. Surface seawater was collected from a running seawater tank at the Ruđer Bošković Institute for Marine Research in October 2014. Deep seawater was sampled with Niskin bottles at ~ 500‐m depth in the Pacific, at ~ 2000‐m depth in the Atlantic and at ~ 2500‐m depth in the Southern Ocean on several cruises between 2016 and 2018 (Table S1). Duplicate incubations with radiolabeled leucine were performed using the complete sampling line of the ISMI as well as detached ISMI bottles. In general, rates measured in the complete ISMI setup were not significantly different from incubations in single detached bottles (Fig. 5; Wilcoxon signed rank test, *n* = 8, *p* = 0.62), suggesting that shear forces due to the diameter of the tubing and valves are not affecting the activity measurements. In addition, the measured leucine incorporation rates obtained with the ISMI bottles were not significantly different from those obtained with polypropylene centrifuge tubes (Wilcoxon signed rank test, *n* = 28, *p* = 0.05; Table S2).

### Performance of ISMI under hydrostatic pressure conditions

The mechanical performance of ISMI at different hydrostatic pressure conditions was tested in two experiments in the pressure tank of the Royal Netherlands Institute for Sea Research (NIOZ) in April 2017. The pressure tank has a diameter of 55 cm and a depth of 200 cm, is filled with freshwater and able to produce pressures of up to 70 MPa. The ISMI was prepared as described above and ^3^H‐leucine was injected into the inlet of the incubation bottles (26 μCi per bottle, in total three bottles) to check for potential leakage of the system under high hydrostatic pressure conditions. One liter of seawater was filled into a folding bag connected to the inlet tubing of the ISMI preceding the first peristaltic pump. The volume for filling the incubation bottles was set at 780 mL (260 mL × three bottles) and for filling the six fixation bottles the volume was 60 mL. The ISMI and the bag were completely submersed in the water of the pressure tank and the system started after the hydrostatic pressure reached 10 and 20 MPa at 15°C.

After depressurization of the pressure tank and recovering the ISMI, the volumes of seawater left the bag, in the incubation and fixation bottles were measured. All the seawater removed from the bag (771 and 790 mL at 10 and 20 MPa, respectively) was retained in the ISMI and no leakage of the tracer was found by measuring the disintegrations per minute (DPM) in the water of the high‐pressure tank. The ISMI worked according to the programed sampling scheme with a collection volume ranging between 90% and 100% of the set volume in the bottles. These results indicate that there is no loss of leucine in the ISMI due the tube connection and transfer of sample between bottles under pressurized conditions and that the ISMI is reliably performing under the high‐pressure conditions in the range of 10–20 MPa.

### Heterotrophic prokaryotic production at in situ hydrostatic pressure

**Fig. 6:**
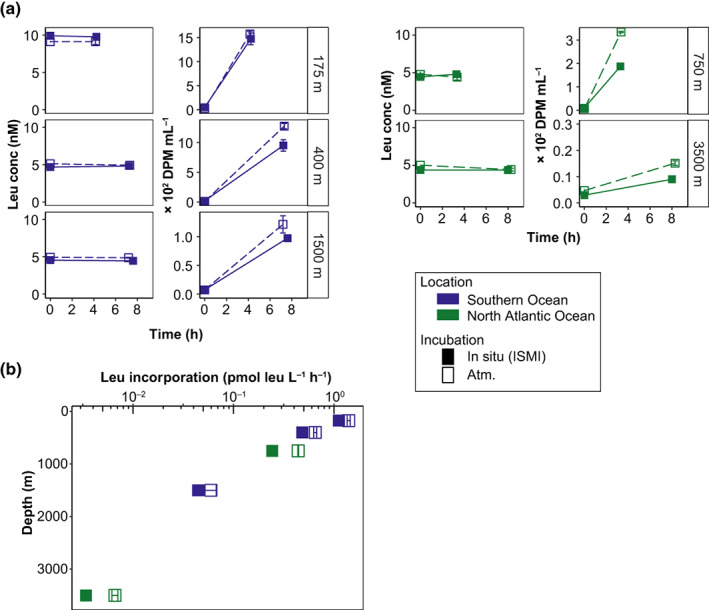
Incubations conducted in the southern and the North Atlantic Ocean under in situ hydrostatic pressure conditions with the ISMI and at atmospheric pressure (atm.) on board of the ships for comparison. (**a**) Concentration and disintegration per minute (DPM) at the start and end of the incubation. The final concentration of leucine was 5 nmol L^−1^, except for the epipelagic sample in the Southern Ocean (10 nmol L^−1^). (**b**) Leucine incorporation rates, error bars indicate SD (*n* = 3) or |mean − replicate| (*n* = 2). Colors indicate the different oceanic regions. Filled and open squares represent in situ (ISMI) and atmosperic pressure incubations, respectively.

A major field study was conducted with the ISMI down to 4000 m depth during several research cruises in the Atlantic, Pacific, and Southern Ocean (Amano et al. 2022). The ISMI worked precisely according to the programed schedule with the expected collection volume of seawater. The success rate of in situ incubations with the ISMI was overall ~ 75% of a total of 38 deployments. In the deployments conducted from 10 to 4000 m depths where half of them were > 2000 m, the success rate of the ISMI itself was 78%. Representative data from epi‐, meso‐, and bathypelagic depths of the North Atlantic off the northwestern Iberian Peninsula and of the Southern Ocean off the Kerguelen islands are shown in Fig. 6. In general, there was some variability in the DPMs among the biological replicates (CV; mean ± SD, *n* = 10, 0.06 ± 0.04 for the atmospheric pressure and 0.08 ± 0.04 for the in situ pressure conditions; Fig. 6a) and of leucine concentrations (CV; 0.03 ± 0.03 for the atmospheric pressure and 0.01 ± 0.01 for the in situ pressure conditions; Fig. 6a). The leucine concentration at T0 was not significantly different from that after the incubation period (Wilcoxon signed rank test, *n* = 10, *p* = 0.232) and the DPMs in the formaldehyde‐killed controls of the ISMI were similar to the killed controls under atmospheric pressure incubations (Wilcoxon signed rank test, *n* = 10, *p* = 0.19), indicating that reproducible results are obtained using ISMI in the field. Concurrently, leucine uptake kinetics were determined on selected samples during the cruises (Fig. S6), revealing that a final leucine concentration of 5 and 10 nmol L^−1^ represents the saturating substrate concentrations depending on the sampling depth.

In the Southern Ocean, leucine incorporation rates under in situ hydrostatic pressure conditions were 1.12 ± 0.04 pmol leu L^−1^ h^−1^ in the epipelagic, 0.48 ± 0.05 pmol leu L^−1^ h^−1^ in the mesopelagic, and 0.04 ± 0.00 pmol leu L^−1^ h^−1^ in the bathypelagic layers (*n* = 2, mean ± |mean − replicate|; Fig. 6b; Table S3). These rates were 67–83% of that measured under atmospheric pressure conditions on samples collected by Niskin bottles (Christaki et al. 2021; Amano et al. 2022). In the North Atlantic, leucine incorporation rates under in situ pressure conditions were 0.24 ± 0.01 pmol leu L^−1^ h^−1^ in the mesopelagic and 0.003 ± 0.000 pmol leu L^−1^ h^−1^ in the bathypelagic layers (Fig. 6b; Table S3), 51–57% of that measured under atmospheric pressure conditions (Amano et al. 2022). Hence, our results indicate substantially lower bulk heterotrophic activity under in situ hydrostatic pressure than under depressurized conditions.

## Discussion

The dark ocean, making up in terms of volume > 90% of the world's living space, is a key ecosystem in biodiversity and functioning of the ocean (Robison 2009; Levin and Le Bris 2015). However, estimates on the metabolic activity of the most abundant living entity in the oceans, the prokaryotes, at depth below 200 m are still relatively rare compared to the data available for epipelagic waters (Herndl et al. 2022). In addition, below those epipelagic waters almost all metabolic activity measurements taken were carried out using decompressed samples. One of the main reasons might be the lack of adequate sampling techniques and the scarcity of instruments allowing to perform activity measurements of prokaryotes under in situ conditions. With the development of the ISMI we addressed this gap and designed a sampler that allows incubating seawater at depth of 4000 m and is both, versatile and easy to handle.

One of the challenging tasks in assessing microbial activity in the dark ocean under in situ pressure conditions is to find adequate material that provides prokaryotic rates comparable to other incubation material used more routinely in labs and withstands the harsh conditions of the deep sea. In our material test experiments, where we measured heterotrophic production at atmospheric pressure conditions, the combination of silicon tubing and polycarbonate bottles produced reliable data without any indication of biases. The ISMI can accurately sample from 50 mL up to tens of liters of seawater, however, it is particularly the lower end of the sampling volume that lends itself for experiments with radiolabeled substrates because radioactive tracers are expensive.

Not all cells respond in the same way to hydrostatic pressure and a subset of the prokaryotic community might show higher activity at increased pressures while others grow optimally under atmospheric conditions but can also grow at bathypelagic conditions, albeit at lower rates than under atmospheric pressure (Bartlett et al. 1995; Yayanos 1995; Fang et al. 2010). Using radiolabeled substrates for microautoradiography combined with fluorescence in situ hybridization, the ISMI can be used for a targeted assessment of the activity of piezophilic, piezotolerant and piezosensitive prokaryotic communities encountered at depth.

To determine the influence of hydrostatic pressure on deep‐sea prokaryotes, in situ prokaryotic activity is typically compared to conventional Niskin sampling and measurements under depressurized conditions. Temperature has a strong influence on the metabolic activity of prokaryotes (Kirchman et al. 2009). Depending on the sampling location, Niskin bottles are occasionally hoisted through a layer of substantially higher temperature than that of meso‐ or bathypelagic waters. We tried to keep the time between sampling from the Niskin bottles and incubation for prokaryotic biomass production at a minimum (10–20 min), yet, samples taken in mid‐latitude bathypelagic waters (~ 2°C) increase in temperature by up to 6°C prior to incubating the samples on board. To bring the temperature back to the in situ temperature for incubations, we used a high precision water bath able to keep the temperature within a range of ±0.04°C of the in situ temperature (Lauda RE120; Germany).

The frequently used in situ incubation device SID [recently modified versions: 4 L‐SID, Bombar et al. (2015); MS‐SID, Edgcomb et al. (2016) and Pachiadaki et al. (2016)] has a single incubation chamber (either 2 or 4 L) which can be flushed with surrounding seawater several times in situ allowing consecutive sampling. The SID can be used in mooring and free‐drifting deployments with time series sampling, whereas the ISMI allows obtaining biological replicates at a specific depth. The incubation chamber of the SID is mainly made of glass (a cylinder part, inside silane treated), which allows also determining the concentrations of some chemical parameters.

Pressure retaining samplers such as the high‐pressure bottles (HPBs) and high‐pressure sampler unit are also used to study deep sea microbes and their activity under in situ pressure conditions (Bianchi et al. 1999; Tamburini et al. 2003; Garel et al. 2019). The high‐pressure retaining serial sampler is operating to a depth of 6000 m (Garel et al. 2019). HPBs are made of titanium or stainless steel (coated with polyetheretherketone) and can be cleaned with acid and autoclaved (Tamburini et al. 2009). For sampling, seawater is distributed through a stainless steel tube trigged by a magnetic valve. For field experiments, the pressure retaining samplers are designed to be mounted on a Niskin rosette frame and processed on board in a lab‐container (Garel et al. 2019). As the ISMI is compact in size and flexible for deployments, it can also be deployed from a small ship (Fig. S1e), mounted on the Niskin rosette frame (Fig. S1a) or attached to the winch cable together with another device (e.g., in situ pump; Fig. S1b).

## Comments and recommendations

The ISMI allows the incubation and fixation of water samples with variable volumes in situ. The incubation bottles can be replaced by 10 L bags to allow collecting a sufficient sample volume for other analyses. If the sampling bags are supplemented with a substrate (e.g., amino acids), the complete mixing with the incoming seawater needs to be tested, similar to our approach described herein. If the ISMI is not deployed in the course of a regular CTD cast, we recommend to attach logging sensors for accurate depth and temperature determination as hydrowires generally do not hang vertically if rolled out for several 1000 m. Due to the relatively small size and low weight of the instrument it is possible to deploy several incubators at different depths, similar to deployments of in situ pumps.

Measuring prokaryotic activity at in situ conditions is still not common practice, consequently only a rather limited number of in situ rates are available to date. Since the ISMI is fairly inexpensive to fabricate and easy to handle it should help to increase the number of in situ measurements of microbial activity and thereby advance our knowledge on the metabolic activity in the ocean's interior.

## Supporting information


**Fig. S1.** Deployment of ISMI mounted on Niskin rosette frame (**a**) and attached on a ship's winch cable (**b**). Incubation with 10 L folding bags (**c**) and a 12 L titanium tank (**d** and **e**) are also possible. The ISMI deployed from a 17 m fishing vessel (**e**).

**Fig. S2.** CAD image of ISMI sampling bottle.

**Fig. S3.** CAD image of rosette tubing clamp unit.

**Fig. S4.** Programming of ISMI. An example of prepared setting sheet (**a**). Blue filled boxes are the data needed as input in the ROCS‐com software. Wiring scheme showing the connections of the electronic parts of the ISMI (**b**). All underwater connectors are rubber molded bulkhead connectors (Seacon). The PC is operated on a Microsoft Windows system and is linked to the controller/communications unit with an RS‐232 cable.

**Fig. S5.** Distribution of a dye‐solution of variable density prepared with artificial seawater (ASW) of a density of 1.03 g cm^−3^.Horizontal (**a**, **b**) and vertical (**g**) setup of ISMI detached bottles. Fifty mL syringes were used to visualize the dye‐solution by adjusting the flow rate to the same volumetric flux as ISMI detached bottles (**c–f**). Δd = (dye‐solution) – (ASW) in g cm^−3^.

**Fig. S6.** ^3^H‐leucine uptake kinetics determined on samples collected from several depths in the Southern Ocean (**a**) and North Atlantic Ocean (**b**). In the Southern Ocean samples, saturating substrate concentrations were reached below 5 nmol L^−1^ leucine and in the north Atlantic among 5–10 nmol L^−1^ leucine. Error bars indicate |mean – replicate| (*n* = 2).

**Table S1.** Locations, date and temperature and salinity of samples used in this study.

**Table S2.** Comparison of leucine incorporation rates (pmol leu L^−1^ h^−1^) from samples incubated in detached ISMI bottles and polypropylene centrifuge tubes. Incubations were conducted at in situ temperature under atmospheric pressure conditions. Numbers indicate mean ± SD of *n* = 3 (detached ISMI bottles) or |mean – replicate| of *n* = 2 (centrifuge tubes).

**Table S3.** Leucine incorporation rates (pmol leu L^−1^ h^−1^) of representative samples incubated at atmospheric pressure (atm.) and in situ pressure conditions.

## Data Availability

Data are available in Tables [Link], [Link], [Link].
