# Supplementary figures and images for: A device for assessing microbial activity under ambient hydrostatic pressure: The in situ microbial incubator (ISMI)
Source: Limnol Oceanogr Methods. 2022 Dec 14;21(2):69–81. doi: 10.1002/lom3.10528 (PMC10946486; doi:10.1002/lom3.10528)

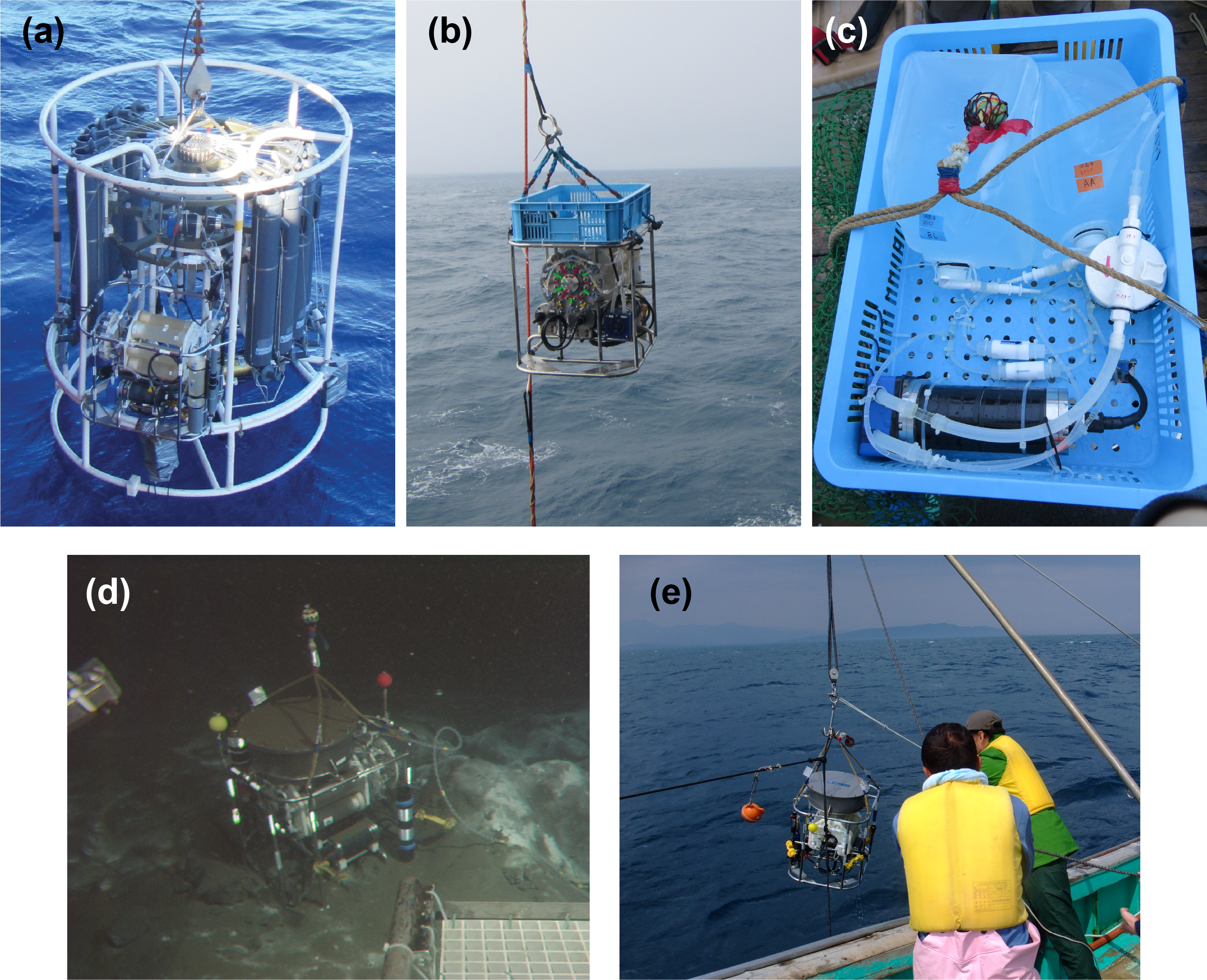

Supplement: Supplementary file 1 — Fig. S1. Deployment of ISMI mounted on Niskin rosette frame (a) and attached on a ship's winch cable (b). Incubation with 10 L folding bags (c) and a 12 L titanium tank (d and e) are also possible. The ISMI deployed from a 17 m fishing vessel (e). [file LOM3-21-69-s002.tif]

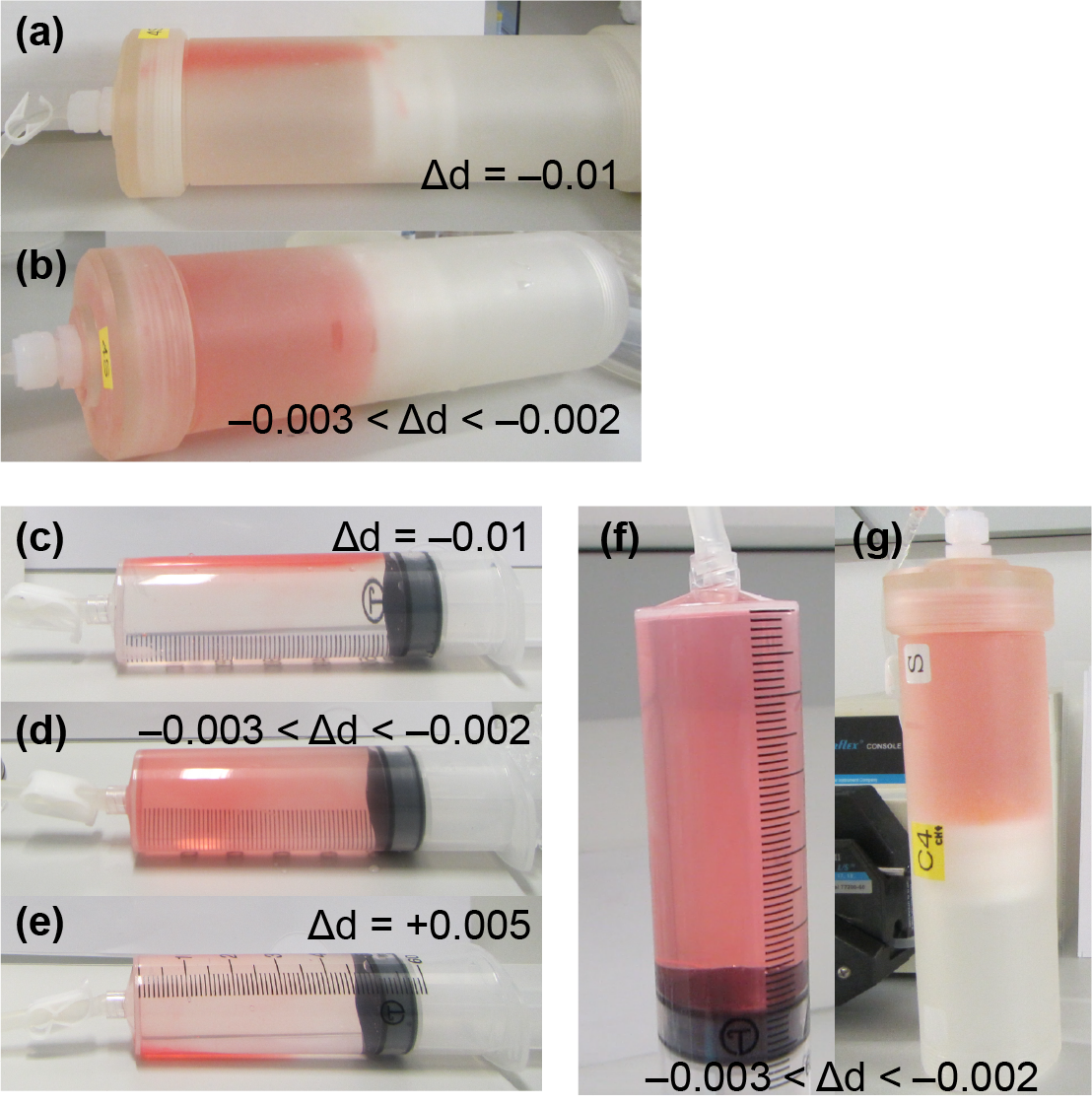

Supplement: Supplementary file 5 — Fig. S5. Distribution of a dye‐solution of variable density prepared with artificial seawater (ASW) of a density of 1.03 g cm−3.Horizontal (a, b) and vertical (g) setup of ISMI detached bottles. Fifty mL syringes were used to visualize the dye‐solution by adjusting the flow rate to the same volumetric flux as ISMI detached bottles (c–f). Δd = (dye‐solution) – (ASW) in g cm−3. [file LOM3-21-69-s005.tif]

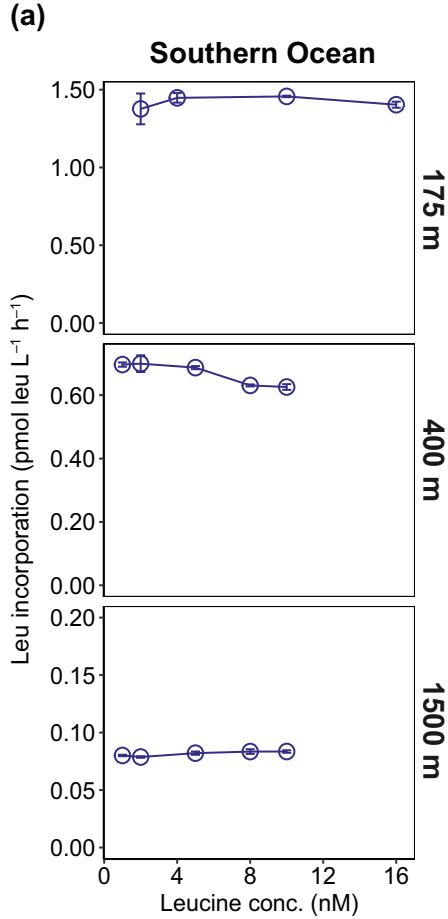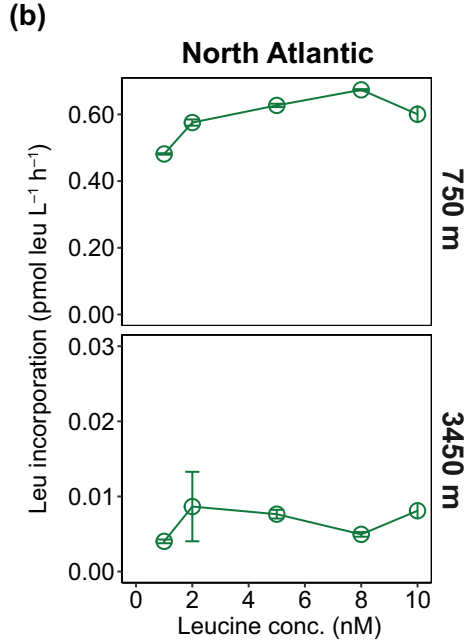

Supplement: Supplementary file 6 — Fig. S6. 3H‐leucine uptake kinetics determined on samples collected from several depths in the Southern Ocean (a) and North Atlantic Ocean (b). In the Southern Ocean samples, saturating substrate concentrations were reached below 5 nmol L−1 leucine and in the north Atlantic among 5–10 nmol L−1 leucine. Error bars indicate |mean – replicate| (n = 2). [file LOM3-21-69-s009.pdf]
