# Supplementary material for: A device for assessing microbial activity under ambient hydrostatic pressure: The in situ microbial incubator (ISMI)
Source: Limnol Oceanogr Methods. 2022 Dec 14;21(2):69–81. doi: 10.1002/lom3.10528 (PMC10946486; doi:10.1002/lom3.10528)

(a)

Volume setting (2 pumps incubation mode)

Total incubation vol		mL

Port 1	Drain		mL	Port 2	Sampling		mL
Port 3	Drain		mL	Port 4	Sampling		mL
Port 5	Drain		mL	Port 6	Sampling		mL
Port 7	Drain		mL	Port 8	Sampling		mL
Port 9	Drain		mL	Port 10	Sampling		mL
Port 11	Drain		mL	Port 12	Sampling		mL

Date and time setting (yyyy/mm/dd, hh:mm)

	**Date**	**Time**
No. 1		
No. 2		
No. 3		
No. 4		
No. 5		
No. 6		

(b)

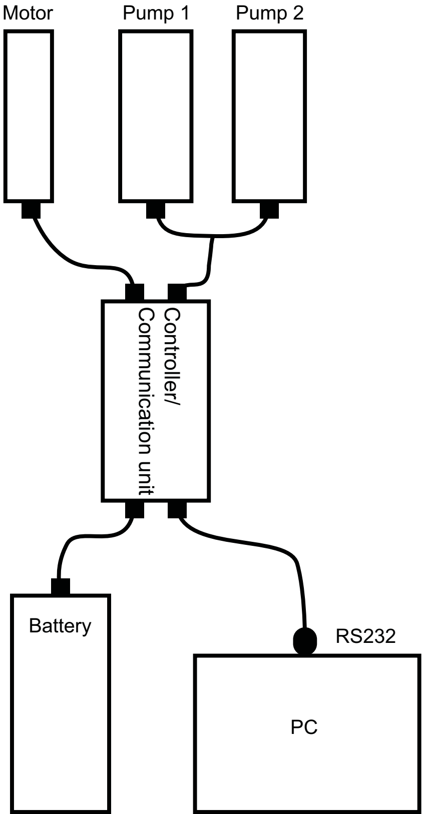

Supplement: Supplementary file 4 — Fig. S4. Programming of ISMI. An example of prepared setting sheet (a). Blue filled boxes are the data needed as input in the ROCS‐com software. Wiring scheme showing the connections of the electronic parts of the ISMI (b). All underwater connectors are rubber molded bulkhead connectors (Seacon). The PC is operated on a Microsoft Windows system and is linked to the controller/communications unit with an RS‐232 cable. [file LOM3-21-69-s001.pdf]
