# Supplementary material for: A device for assessing microbial activity under ambient hydrostatic pressure: The in situ microbial incubator (ISMI)
Source: Limnol Oceanogr Methods. 2022 Dec 14;21(2):69–81. doi: 10.1002/lom3.10528 (PMC10946486; doi:10.1002/lom3.10528)
Supplement: Supplementary file 7 — Table S1. Locations, date and temperature and salinity of samples used in this study. [file LOM3-21-69-s007.docx]

| **Cruise/**  **Location** | **Area** | **St** | **Date** | **Lat (°)** | | **Long (°)** | | **Depth** | **Temp** | **Sal** |
| --- | --- | --- | --- | --- | --- | --- | --- | --- | --- | --- |
|  |  |  |  |  |  |  |  | **(m)** | **(°C)** |  |
| NT1005^a^ | Kagoshima Bay | N/A | 19 May 10 | 31.6679 | N | 130.7615 | E | 198 | 16.34 | 34.10 |
| Yaizu^a^ | Suruga Bay | N/A | 05 Jul 10 | 34.8046 | N | 138.5512 | E | 400 | 8 | N/D |
| Yaizu^b^ | Suruga Bay | N/A | 29 Sep 13 | 34.8535 | N | 138.3972 | E | 400 | 6 | N/D |
| Rovinj^c,d^ | Adriatic Sea | N/A | 24 Oct 14 | 45.0856 | N | 13.6394 | E | 20 | 19.5 | N/D |
| Rovinj^c,d^ | Adriatic Sea | N/A | 25 Oct 14 | 45.0856 | N | 13.6394 | E | 20 | 19.7 | N/D |
| SO248^d^ | Pacific | 17 | 29 May 16 | 54.0013 | N | 179.5813 | E | 501 | 3.63 | 34.08 |
| NIOZ jetty^e^ | Wadden Sea | N/A | 13 Apr 17 | 53.0018 | N | 4.7892 | E | Surface | 10 | N/D |
| NIOZ jetty^e^ | Wadden Sea | N/A | 14 Apr 17 | 53.0018 | N | 4.7892 | E | Surface | 10 | N/D |
| M139^d^ | Atlantic | A1 | 12 Jul 17 | 15.8860 | N | 68.9148 | W | 2002 | 4.12 | 34.98 |
| M139 | Atlantic | A3 | 22 Jul 17 | 23.5539 | N | 48.0839 | W | 2000 | 3.68 | 35.00 |
| M139^c^ | Atlantic | A3 | 22 Jul 17 | 23.5539 | N | 48.0839 | W | 3001 | 2.72 | 34.93 |
| M139 | Atlantic | A5_6 | 31 Jul 17 | 10.3391 | N | 36.9600 | W | 3999 | 2.33 | 34.89 |
| M139 | Atlantic | A5_6 | 31 Jul 17 | 10.3393 | N | 36.9618 | W | 474 | 8.56 | 34.90 |
| MOB | Southern | M2_1 | 26 Feb 18 | 50.6160 | S | 72.0011 | E | 448 | 2.25 | 34.38 |
| MOB | Southern | M4_1 | 02 Mar 18 | 52.6003 | S | 67.1999 | E | 3998 | 0.16 | 34.67 |
| MOB | Southern | M3_1 | 03 Mar 18 | 50.6835 | S | 68.0621 | E | 1499 | 2.00 | 34.76 |
| MOB^f^ | Southern | M2_2 | 06 Mar 18 | 50.6251 | S | 72.0137 | E | 400 | 2.16 | 34.30 |
| MOB^d^ | Southern | M1 | 08 Mar 18 | 49.8502 | S | 74.9017 | E | 2499 | 1.01 | 34.72 |
| MOB | Southern | M4_2 | 12 Mar 18 | 52.6014 | S | 67.2010 | E | 3500 | 0.50 | 34.69 |
| MOB^f^ | Southern | M2_3 | 16 Mar 18 | 50.6159 | S | 72.0013 | E | 175 | 1.72 | 33.99 |
| MOB^f^ | Southern | M3_3 | 18 Mar 18 | 50.6877 | S | 68.0664 | E | 1498 | 2.07 | 34.76 |
| RadProf18 | North Atlantic | 12 | 21 Aug 18 | 43.0000 | N | 10.1520 | W | 2759 | 2.86 | 34.95 |
| RadProf18^f^ | North Atlantic | 111 | 24 Aug 18 | 42.9998 | N | 14.0350 | W | 3443 | 2.63 | 34.93 |
| RadCan18^f^ | North Atlantic | C3 | 27 Aug 18 | 43.7668 | N | 6.1672 | W | 743 | 10.23 | 35.67 |
| RadCan18 | North Atlantic | S7 | 28 Aug 18 | 43.8003 | N | 3.7830 | W | 2220 | 3.51 | 35.00 |
| RadCan18^g^ | North Atlantic | G4 | 29 Aug 18 | 44.3330 | N | 5.6665 | W | 3930 | 2.49 | 34.91 |

St: station

N/A: not applicable

N/D: not determined

MOB: MOBYDICK, RadProf18: RADPROF201808, RadCan18: RADCAN201808

^a^12L incubation tank

^b^10L incubation bag

^c^Comparison between ISMI incubation bottles and commercially available containers

^d^Tests comparing complete ISMI setup and ISMI detached bottles

^e^Experiment using the high pressure tank at the NIOZ. Sea surface water collected with a bucket.

^f^Incubation under in situ vs on-deck atmospheric pressure conditions

^g^Experiment on mixing of substrate in the ISMI detached bottles
