# Supplementary material for: A device for assessing microbial activity under ambient hydrostatic pressure: The in situ microbial incubator (ISMI)
Source: Limnol Oceanogr Methods. 2022 Dec 14;21(2):69–81. doi: 10.1002/lom3.10528 (PMC10946486; doi:10.1002/lom3.10528)
Supplement: Supplementary file 8 — Table S2. Comparison of leucine incorporation rates (pmol leu L−1 h−1) from samples incubated in detached ISMI bottles and polypropylene centrifuge tubes. Incubations were conducted at in situ temperature under atmospheric pressure conditions. Numbers indicate mean ± SD of n = 3 (detached ISMI bottles) or |mean – replicate| of n = 2 (centrifuge tubes). [file LOM3-21-69-s004.docx]

| **Cruise** | **St** | **Depth (m)** | **Leu incorporation rate** | | **A/B*** |
| --- | --- | --- | --- | --- | --- |
|  |  |  | **Detached ISMI bottle (A)** | **Centrifuge tube (B)** |  |
| M139 | A5_6 | 474 | 0.34 ± 0.02 | 0.30 ± 0.00 | 1.1 |
| M139 | A5_6 | 3999 | 0.03 ± 0.00 | 0.02 ± 0.00 | 1.5 |
| MOB | M1 | 2499 | 0.15 ± 0.01 | 0.16 ± 0.01 | 1.0 |
| MOB | M2_1 | 448 | 1.23 ± 0.02 | 1.35 ± 0.00 | 0.9 |
| MOB | M2_2 | 400 | 0.65 ± 0.02 | 0.69 ± 0.00 | 0.9 |
| MOB | M2_3 | 175 | 1.40 ± 0.05 | 1.46 ± 0.00 | 1.0 |
| MOB | M3_1 | 1499 | 0.07 ± 0.00 | 0.08 ± 0.00 | 0.9 |
| MOB | M3_3 | 1498 | 0.06 ± 0.01 | 0.08 ± 0.00 | 0.7 |
| MOB | M4_1 | 3998 | 0.02 ± 0.00 | 0.02 ± 0.00 | 1.1 |
| MOB | M4_2 | 3500 | 0.01 ± 0.00 | 0.02 ± 0.00 | 0.7 |
| RadProf18 | 12 | 2759 | 0.01 ± 0.00 | 0.01 ± 0.00 | 1.0 |
| RadProf18 | 111 | 3443 | 0.01 ± 0.00 | 0.01 ± 0.00 | 0.7 |
| RadCan18 | C3 | 743 | 0.44 ± 0.00 | 0.49 ± 0.03 | 0.9 |
| RadCan18 | S7 | 2220 | 0.03 ± 0.00 | 0.04 ± 0.01 | 0.7 |

*A/B: Ratio of leucine incorporation rate measured in ISMI bottle to centrifuge tubes

St: station, MOB: MOBYDICK, RadProf18: RADPROF201808, RadCan18: RADCAN201808
