# Supplementary material for: A device for assessing microbial activity under ambient hydrostatic pressure: The in situ microbial incubator (ISMI)
Source: Limnol Oceanogr Methods. 2022 Dec 14;21(2):69–81. doi: 10.1002/lom3.10528 (PMC10946486; doi:10.1002/lom3.10528)
Supplement: Supplementary file 9 — Table S3. Leucine incorporation rates (pmol leu L−1 h−1) of representative samples incubated at atmospheric pressure (atm.) and in situ pressure conditions. [file LOM3-21-69-s003.docx]

| **Location** | **Cruise** | **St.** | **Depth (m)** | **In situ** | | |  | **Atm.** | | | **ref.** |
| --- | --- | --- | --- | --- | --- | --- | --- | --- | --- | --- | --- |
|  |  |  |  | **mean** | **\|mean – replicate\|** | **n** |  | **mean** | **SD** | **n** |  |
| Southern Ocean | MOB | M2_2 | 400 | 0.484 | 0.048 | 2 |  | 0.651 | 0.023 | 3 | Amano et al. 2022  https://doi.org/10.1038/s41561-022-01081-3 |
|  | MOB | M2_3 | 175 | 1.118 | 0.038 | 2 |  | 1.400 | 0.050 | 3 |  |
|  | MOB | M3_3 | 1500 | 0.044 | 0.002 | 2 |  | 0.060 | 0.008 | 3 |  |
| North Atlantic | RadProf18 | 111 | 3500 | 0.003 | 0.000 | 2 |  | 0.007 | 0.000 | 3 |  |
|  | RadCan18 | C3 | 750 | 0.243 | 0.006 | 2 |  | 0.440 | 0.004 | 3 |  |

MOB: MOBYDICK, RadProf18: RADPROF201808, RadCan18: RADCAN201808.
